# COVID-19 pandemic and first episode of psychosis: Clinical characteristics

**DOI:** 10.1192/j.eurpsy.2021.1793

**Published:** 2021-08-13

**Authors:** L. Brahmi, H. Ben Ammar, G. Hamdi, E. Khelifa, R. Felhi, L. Mnif

**Affiliations:** Psychiatry Department, Razi hospital, manouba, Tunisia

**Keywords:** psychotic disorder, COVID-19, pandemic, delusions

## Abstract

**Introduction:**

The rapid spread of the SARS‐CoV‐2 pandemic among the world poses challenges to the management of both physical and mental health. This unexpected situation could predict an exacerbation of anxiety, depressions, obsessions, and even multiple cases of psychosis.

**Objectives:**

The aim of this literature review is to identify and analyze studies conducted in 2020 that investigate the incidence of psychotic disorders, related to COVID-19 pandemic and describe its symptoms.

**Methods:**

A systematic search in the PubMed electronic database was performed using keywords “COVID-19”, “pandemics”, “psychotic symptoms”, and “ first episode of psychosis” Relevant literature was limited to articles describing studies conducted and published in 2020.

**Results:**

9 papers met the inclusion criteria. The selected studies reported 20 cases of psychosis in patients with no psychiatric history, directly triggered by stress derived from the COVID-19 pandemic and by social distancing and quarantine. All cases were characterized by sudden behavioral changes out of character, increased concern about coronavirus risk infection, anxiety, psychomotor agitation, and insomnia. In multiple cases, psychotic symptoms were characterized by thoughts of reference, persecution, and structured delusional. 5 patients were convinced that COVID-19 Pandemic was part of a conspiracy and that someone was trying to infect them by diffusing the COVID-19 or other pollutants. Half of the patients had the delusional conviction that they got infected and they were contagious.

**Conclusions:**

COVID-19 pandemic appears to be the trigger for precipitating psychosis which has a high risk of suicidal behavior. During pandemics, mental health professionals should carry out more focused diagnostic and therapeutic strategies.

**Disclosure:**

No significant relationships.

